# Developing a Novel Ontology for Cybersecurity in Internet of Medical Things-Enabled Remote Patient Monitoring

**DOI:** 10.3390/s24092804

**Published:** 2024-04-27

**Authors:** Kulsoom S. Bughio, David M. Cook, Syed Afaq A. Shah

**Affiliations:** School of Science, Edith Cowan University, 270 Joondalup Dr, Joondalup, WA 6027, Australia; d.cook@ecu.edu.au (D.M.C.); afaq.shah@ecu.edu.au (S.A.A.S.)

**Keywords:** Internet of Medical Things (IoMT), MIoT (Medical Internet of Things) Ontology, knowledge organization system (KOS), ontology development, resource description framework (RDF), first-order logic (FOL), description logic (DL), Web Ontology Language (OWL)

## Abstract

IoT has seen remarkable growth, particularly in healthcare, leading to the rise of IoMT. IoMT integrates medical devices for real-time data analysis and transmission but faces challenges in data security and interoperability. This research identifies a significant gap in the existing literature regarding a comprehensive ontology for vulnerabilities in medical IoT devices. This paper proposes a fundamental domain ontology named MIoT (Medical Internet of Things) ontology, focusing on cybersecurity in IoMT (Internet of Medical Things), particularly in remote patient monitoring settings. This research will refer to similar-looking acronyms, IoMT and MIoT ontology. It is important to distinguish between the two. IoMT is a collection of various medical devices and their applications within the research domain. On the other hand, MIoT ontology refers to the proposed ontology that defines various concepts, roles, and individuals. MIoT ontology utilizes the knowledge engineering methodology outlined in Ontology Development 101, along with the structured life cycle, and establishes semantic interoperability among medical devices to secure IoMT assets from vulnerabilities and cyberattacks. By defining key concepts and relationships, it becomes easier to understand and analyze the complex network of information within the IoMT. The MIoT ontology captures essential key terms and security-related entities for future extensions. A conceptual model is derived from the MIoT ontology and validated through a case study. Furthermore, this paper outlines a roadmap for future research, highlighting potential impacts on security automation in healthcare applications.

## 1. Introduction

In the era of Industry 4.0, Internet of Things (IoT) has rapidly expanded, particularly in the healthcare sector, where Internet of Medical Things (IoMT) has emerged as a specialized branch. IoMT focuses on integrating interconnected medical devices and information systems, revolutionizing healthcare by enabling real-time data collection, analysis, and transmission [1]. This technology offers opportunities for remote patient monitoring, improved medical processes, and improved decision-making.

IoMT operates through a network of connected medical devices, sensors, and information systems, facilitating the collection and transmission of continuous automatic and health data. Various devices, such as vital signs monitors, medication delivery tools, fitness trackers, and home monitoring devices, contribute to this interconnected system. Data collected can be transmitted to healthcare professionals, hospitals, or cloud-based systems for analysis and storage, leading to innovative medical applications such as the remote monitoring of chronic patients and personalized healthcare, eventually advancing diagnosis and treatment in the healthcare industry [2,3].

Despite the advances and expansion of Internet of Medical Things (IoMT) technology, numerous challenges must be faced to ensure its widespread success and adoption. These challenges encompass issues such as data security and privacy, ensuring the interoperability of devices and systems, efficient data management, and addressing the long-term sustainability of technology. Knowledge organization systems (KOSs) are one of the solutions used to organize and categorize information, knowledge, and data, making it more accessible, searchable, and understandable in the field of IoMT. These systems help individuals and organizations efficiently manage and retrieve information. Various types of knowledge organization systems exist, including taxonomies, thesauri, ontologies, and controlled vocabularies, among others [4]. The choice of a particular KOS depends on the nature and requirements of the information and the users. For example, a taxonomy might be sufficient for simple categorization tasks, while an ontology would be better suited for complex data analysis and Semantic Web applications.

### 1.1. The Need for a Cybersecurity Ontology for Remote Patient Monitoring

In the dynamic field of healthcare technology, especially in remote patient monitoring (RPM), the importance of robust cybersecurity measures has grown significantly. With medical devices becoming increasingly interconnected and reliant on networked systems, the threat of cybersecurity risks such as data breaches and device vulnerabilities has become more pronounced [5]. In this situation, maintaining cyber-resilience across networks poses significant challenges, particularly concerning data security, scalability, heterogeneity, interoperability, and integration. These difficulties arise from the exposure of data and the diverse functionalities and specifications of various devices within the network [6]. Hence, there is a crucial need for methods and tools to promptly detect data breaches and respond effectively to identified cyberattacks. In this regard, ontology is a formal representation of knowledge or concepts within a specific domain [7].

The primary benefit of ontologies lies in interoperability. By establishing a shared vocabulary, data can be shared and exposed using a formal definition, enabling others to understand and utilize it effectively. This facilitates the development of applications that consume the data with interoperability and RDF (resource description framework), forming the foundation of the Semantic Web concept. Secondly, ontologies support inferencing, where fragments of knowledge within the ontology can be used to obtain new perceptions/knowledge. This process involves inferring new knowledge based on existing data, allowing for the derivation of additional facts from existing data by using ontological principles [8].

This paper proposes a fundamental domain ontology named MIoT ontology, focusing on cybersecurity in IoMT, especially in remote patient monitoring settings. This research will refer to two closely related acronyms that may appear similar. It is important to distinguish between the two terms used in this research: IoMT (Internet of Medical Things) and MIoT (Medical Internet of Things) ontology. IoMT refers to the network of interconnected medical devices and applications that collect and transmit health data over the Internet for monitoring, diagnosis, and treatment purposes. On the other hand, MIoT ontology specifically relates to the structured representation and organization of knowledge within the domain of medical IoT, focusing on the semantics and relationships of medical devices and vulnerabilities. The development of an MIoT ontology would provide a structured framework for organizing and categorizing cybersecurity-related concepts such as vulnerability, exploit, and attack, defining their relationship within the RPM ecosystem. By clearly defining the key concepts and their connections, this ontology would empower healthcare providers and cybersecurity professionals to better comprehend, evaluate, and mitigate potential vulnerabilities to patient data and device security.

### 1.2. The Role of Semantic Models in the Internet of Medical Things

A semantic model is a high-level, abstract representation of data that focuses on capturing and conveying meaning rather than just structure or syntax, allowing interpretation from instances. In data integration and exchange scenarios, semantic models ensure effective understanding and integration of data from different sources, such as medical sensors. By encoding meaning and relationships between data elements, semantic models enable interoperability between systems, providing a structured framework for organizing meaningful relationships, as demonstrated by RDF triples in Semantic Web data.

The Semantic Web, an extension of the World Wide Web (W3C) [9], aims to make web content more machine-understandable using standard semantic technologies. Its success relies on the quality of its underlying ontologies, which provide a shared understanding of a domain for communication between people and heterogeneous systems. Ontologies facilitate interoperability by establishing a common understanding of concepts and relationships, crucial for data integration and semantic interoperability. They also aid in retrieving heterogeneous information from cross-domain applications. Semantic web technologies, including RDF, OWL, and XML, are utilized to construct ontologies for different domains [10].

Our research paper aims to develop a fundamental ontology for the IoMT domain; we named it the MIOT (Medical Internet of Things) ontology, focusing on essential terms related to the cybersecurity domain. To achieve this, we employ scientific methods and tools in the definition process and subsequently demonstrate the ontology’s applicability to information systems. The resulting ontology serves as a conceptual ontological model that assesses the potential for establishing semantic interoperability among medical devices from heterogeneous sources.

This paper is organized as follows: the existing work is discussed in Section 2. The ontological technology is introduced in Section 3, followed by a methodology of our research approach in Section 4. This methodology outlines the ontology’s domain and relevant terminology, explores possible interfaces with existing ontologies, and defines important terms. It further shows the hierarchical structure for concepts derived from identified terms, specifying their required properties, followed by formal grounding with description logic. Subsequently, in Section 5, a conceptual model is derived from the ontology and validated through a case study. Finally, Section 6 is presented to discuss challenges and provide potential avenues for future research, followed by Section 7, where we summarize our work.

## 2. Related Work

This work seeks to enhance cyber-resilience in healthcare, where challenges such as interoperability, heterogeneity, scalability, integration, and visualization are dominant. Various standards and frameworks, including SNOMED CT [11], HIPPA [12], HL7 FHIR [13], STIX [14], NIST [15,16], and HITRUST [17], among others, have been developed to address these concerns. For instance, the industry standard National Institute of Standards and Technology (NIST) revised its cybersecurity recommendations for healthcare in mid-2022, aiming to safeguard patients’ health data to uphold the confidentiality, integrity, and availability of health data [18].

At the same time, researchers have responded to these challenges by leveraging semantic models and frameworks. For instance, the researchers in [19] conducted a comprehensive analysis of various technologies, system architectures, optimization factors, and challenges associated with implementing IoT in hospital settings. It serves as a link between business applications and sensors within a unified network, paving the way for the development of an interoperable smart hospital design. However, the research does not address the privacy and security risks associated with the creation of such a hospital design.

Gorrepati et al. [20] proposed an ontology-based modeling framework and IoT-based semantic M2M platform. The framework employs IoT healthcare domain ontologies to design knowledge frameworks, automate sequencing, and provide semantic descriptions of concepts and relationships. The use of SWRL rules helps overcome expressivity limitations in property association, enabling spatial connection reasoning. The constructed system, developed using Protégé and its plug-ins, focuses on enhancing hospital device interoperability and semantic annotation in IoT applications. Sharma et al. [21] proposed an IoT-powered, ontology-based remote access model and bio-wearable sensor system for the early detection of COVID-19, utilizing 1D biomedical signals, which contain PPG (photoplethysmography), ECG (electrocardiogram), accelerometer, and temperature. This ontology-based monitoring system analyzes the challenges around privacy and security issues and is also simulated by using a Cooja simulator to observe the efficiency of the proposed model.

A study proposed by Pazienza et al. [22], named the Digital Future project, where Semantic Web services are combined with service-oriented architecture (SOA)-based principles using an IoE (Internet of Everything) platform to address interoperability at the semantic level, employing middleware components. In a parallel effort, Kotha et al. [23] focused on enhancing interoperability in healthcare IoT devices through a study leveraging natural language processing (NLP) techniques, including bidirectional encoder representations from transformers (BERT) for string matching and a fuzzy inference system (FIS) for data correlation. Although the study primarily aimed to improve data communication efficiency among healthcare IoT devices and demonstrated results to enhance IoT interoperability in healthcare, it did not directly address cyberattack detection.

A comprehensive scoping review was conducted by Luschi et al. [24] to identify and analyze available ontologies capable of representing all relevant use cases describing the hospital environment in relation to the European project ODIN and its future expansion. The review was conducted on the Scopus database in January 2023 using the PRISMA extensions for scoping reviews. Two reviewers screened 3225 documents that resulted from the database search. Finally, they filtered the results to a final set of 32 articles for the analysis of the results. In this scoping review, famous ontologies in the field of healthcare were discussed, such as the SNOMED-CT ontology (SCTO) [25], Dietary Lifestyle Ontology (DILON) [26], and International Classification of Diseases (ICD) [27], among others. However, the researchers solely concentrated on medical-related aspects and neglected security concerns.

In terms of ontology development for cybersecurity, a well-known ontology named the IDS (intrusion detection system) ontology was created by Undercoffer et al. [28]. IDS was extended as the Unified Cybersecurity Ontology (UCO) by More et al. [29] to establish a standardized framework for describing the cybersecurity domain. This initiative seeks to transition cybersecurity standards from mere syntactic representations to more semantically enriched ones. Specifically, the UCO serves as a semantic counterpart to STIX (Structured Threat Information eXpression) [14], incorporating references to external standards such as CVEs (common vulnerabilities and exposures), CWE (common weakness enumeration), and CAPEC (common attack pattern enumeration and classification), among others. UCO provides an overview of general cybersecurity concepts such as potential attacks, attack patterns, methods, etc., but dedicates minimal attention to describing the infrastructure itself.

Following this, Bruno et al. [30] introduced novel concepts such as asset and security mechanisms in the IoTSec ontology, which were not part of UCO [29], thereby providing a detailed overview of the infrastructure. The project’s primary objective was to ensure that companies operating in the Internet of Things sector utilized their devices securely, aiding in the identification of vulnerabilities and critical points. However, a drawback of IoTSec lies in the static nature of its knowledge regarding vulnerability and threat classes. These aspects lack integration with external sources and require direct management and maintenance during ontology implementation. However, these ontologies are IoT-specific, but our work focuses on cybersecurity aspects for IoMT, particularly in a remote patient monitoring setting, where the MIoT ontology captures the semantics related to concepts and relationships for vulnerability detection in medical devices.

The existing literature in the fields of healthcare and cybersecurity highlights a substantial research gap, specifically the absence of a comprehensive ontology that can effectively offer knowledge regarding vulnerabilities in Medical Internet of Things (IoT) devices [24,27,29]. In response, this research endeavors to introduce a novel MIoT ontology that not only addresses this gap but also provides a detailed mechanism for developing practical solutions by incorporating semantics.

## 3. Ontological Technology

Ontology was originally rooted in philosophy, denoting an explanation or description of objective existence, emphasizing the philosophical exploration of being and various types of existence. The term ‘ontology’ or (ontologia) was coined in 1613 independently by two philosophers, Rudolf Gockel and Jacob Lorhard [6]. In the realm of artificial intelligence (AI), Neches et al. [31] introduced the first AI-specific definition, characterizing ontology as the framework defining fundamental terms and relations that constitute the vocabulary of a particular domain, along with rules governing their combination to extend the vocabulary. Gruber T. [7] offered a widely adopted definition, presenting ontology as an explicit ‘Specification of a Conceptualization’.

Pinto and Martins [32] defined ontology as ontologies fostering interoperability among information systems, enabling intelligent processing by agents, and facilitating the sharing and reuse of knowledge across various systems. They establish a shared and unified understanding of a domain, facilitating communication among individuals and heterogeneous and diverse application systems. Generally, ontologies consist of a collection of terms representing concepts organized in a hierarchical structure, along with specifications defining their meanings.

Over time, this understanding gained acceptance among scholars and engineers. In the field of artificial intelligence, the term ‘ontology’ is defined in various ways by different scholars. For this guide, ontology is a knowledge representation (KR) system based on description logics (DLs). Ontology is understood as a formal and explicit representation of concepts within a specific domain. Formal indicates that the ontology is machine-readable, while explicit refers to the constraints applied to concepts [33]. ‘Classes’ are referred to as ‘concepts’). It includes the properties (or ‘slots’, also known as ‘roles’ or ‘properties’) that characterize each concept and the constraints on these properties (termed ‘facets’, also known as ‘role restrictions’). Table 1 shows the difference between first-order logic (FOL), description logic (DL), and Web Ontology Language (OWL) terminology for concept, role, and constant [34].

Numerous ontologies, such as description logic (DL) knowledge bases, have been created to offer comprehensive insights into diverse domains. An ontology comprises an ABox, containing assertion axioms between entities or between a concept and an entity, and a TBox, containing terminology axioms between two concepts [35,36]. An ontology, when combined with specific instances (ABox, i.e., the instances of the model) of these classes (TBox model, i.e., the terminological component), forms what is known as a ‘knowledge base’ [37] and is usually represented in RDF and OWL. The definitions of these terms are given in Table 2. In the DL Knowledge base (KB), TBox is a set of ‘terminological’ axioms and Abox is a set of ‘assertional’ axioms [38].

In this regard, ontologies provide a conceptual framework for organizing knowledge. They define concepts or entities relevant to a specific domain or problem. Ontologies often have a hierarchical structure with broader, more general concepts at the top and narrower, more specific concepts below. This hierarchy allows for the classification and categorization of information.

Ontologies distinguish between classes (abstract concepts or categories) and instances (individual entities belonging to a class). For example, ‘MedicalDevice’ is a class, while ‘BPMonitor’ is an instance of the ‘MedicalDevice’ class. Ontologies specify properties or attributes associated with concepts. These properties describe the characteristics or features of instances. For example, a ‘Vulnerability’ class might have properties like ‘hasCVEID’ and ‘publishedDate’. Ontologies also define relationships between concepts called object properties. These relationships provide context and meaning to the concepts and can be hierarchical (is-a), part-whole (has-part), spatial (located-in), and temporal (preceded-by).

In the context of vulnerability detection in IoMT, ontology provides a structured and organized framework for categorizing, classifying, and searching for information related to vulnerabilities and cyberattacks in IoMT. This structured approach, along with semantics, improves the efficiency and accuracy of vulnerability detection processes and enables better decision making in addressing cybersecurity attacks. For instance, ontology is used to model the relationships between various elements in the cybersecurity domain and remote patient monitoring, including medical devices, connectivity, targets, vulnerabilities, exploits, and affected assets.

## 4. A Proposed Methodology for Ontology Development

Developing an ontology is a collaborative task where domain experts can identify the concepts and define relationships in the domain work with knowledge engineers. Domain ontology is a very important part of our research work where we apply ontology engineering practices to develop the proposed ontology. However, note that there is not any particular or correct way to develop the ontology, and researchers apply different ways to develop the ontology [39,40,41,42]. We utilize the knowledge engineering methodology (KEM) outlined in Ontology Development 101 [37], along with the structured life cycle proposed by Gómez-Pérez et al. [43]. These methodologies were chosen for their comprehensive step-by-step procedures in ontology development.

Ontology Development 101 adopts a process-oriented approach, accommodating setbacks and emphasizing continuous improvement through multiple iterations. This methodology is particularly crucial for our ontology, given its inherent complexity and the need for ongoing extensions due to the dynamic nature of the domain, characterized by frequent changes and constant development. To address this, it is recommended to frame the ontology under the open-world assumption (OWA) [44,45]. This assumption accommodates incomplete information, acknowledging that there might be more pertinent details than are currently available. It proves advantageous for expressing knowledge in a manner that is adaptable and widely employed, particularly within the field of artificial intelligence. Our goal is to develop a domain ontology that allows for future extensions, aligning with the evolving nature of the domain.

The domain ontology named MIoT ontology provides a common understanding between two specific areas, i.e., remote patient monitoring (RPM) and cybersecurity. This cross-domain ontology shows how to detect vulnerabilities in remote patient monitoring systems by using semantic standards and technologies like RDF and OWL. By defining the resources, vocabularies, definitions, annotations, etc., related to medical devices in remote patient monitoring, vulnerabilities are detected that are ultimately exploited by attacks.

The ontology development process is divided into the five following stages shown in Table 3. Specification and scope, existing ontologies reused, term enumeration, conceptualization, and formal grounding with description logics act as a foundation for this research work. This process involves the articulation of the knowledge area, the research’s purpose, and the formulation of concepts, relationships, and axioms. MIoT is an informal ontology [32] where a conceptual model is presented in this paper.

### 4.1. Specification and Scope

Due to advancements in communication networks, the Internet of Things (IoT) has brought new directions in which a person can connect with any device from any location. The remote healthcare patient monitoring system is one example, where IoT devices can detect the patient’s condition and whether they behave normally or not by checking their blood pressure, heart rate, etc. In case of any abnormal reading, it will alert the doctor or caregiver for assessment. While these IoT networks are very beneficial and attractive towards healthcare and the community, at the same time, there are also chances of the occurrence of security risks.

The specification signifies the importance of the ontology development process, which is descriptive and prescriptive [46] and notifies who is going to use the model and why [47]. Given the sensitivity of the healthcare domain to cyberattacks and vulnerabilities due to the exposure of heterogeneous data on the web, the primary goal is to enhance cybersecurity measures, ensuring patient safety and data integrity in remote monitoring environments. The proposed domain ontology seeks to systematically address vulnerabilities in medical devices utilized for remote patient monitoring within the healthcare domain. This involves developing a comprehensive understanding of interconnected concepts relevant to the medical setting, including medical devices containing actuators, medical sensors, their connectivity (e.g., Bluetooth or Wi-Fi), and associations with vulnerabilities, exploits, and supporting systems and networks. It incorporates a well-defined vocabulary (i.e., schema.org), including terms such as medical device, person, product, service, etc. The ontology features a hierarchical structure with defined classes and subclasses, such as person as a class with three subclasses: patient, doctor, and healthcare provider. Importantly, this ontology excludes non-medical IoT devices and vulnerabilities unrelated to healthcare. This phase establishes the foundation for the subsequent ontology development phase, which involves conceptualization.

#### 4.1.1. Data Acquisition

The phase of acquiring knowledge plays a crucial role in the ontology-building approach from the ground up. It ensures the gathering of essential data for the design and population of the ontology. This research collects and observes data and information from diverse sources to ensure that the ontology is comprehensive, well-informed, and reflective of the current state of knowledge in the domain of medical device vulnerabilities, particularly cybersecurity in healthcare in general. This approach enhances the ontology’s ability to address real-world challenges and capture a wide spectrum of information relevant to the identification and management of vulnerabilities in medical devices for remote patient monitoring.

The formal representation of knowledge in a specific domain includes information derived from the specifications of medical devices such as technical details, functionalities, configurations, and any other relevant attributes (i.e., manufacturer information) that define the characteristics of these medical devices. Additionally, data from databases dedicated to cybersecurity e.g., National Vulnerability Database (NVD), is incorporated. These databases likely contain information about known vulnerabilities, threats, attack patterns, and countermeasures related to medical devices and healthcare systems. Information obtained from incident reports document instances of security breaches, cyberattacks, or other adverse events related to medical devices. Analyzing these reports helps in understanding real-world scenarios (i.e., ransomware) and identifying potential vulnerabilities (i.e., Log4J). Moreover, insights from the relevant literature, such as research papers, articles, and publications in the fields of medical device security, cybersecurity, and healthcare technology, are used. This includes academic studies, industry reports, and expert opinions that contribute to the knowledge base.

#### 4.1.2. Competency Questions

(a) What types of devices are utilized in the IoMT environment?
(b) Who are the vendors of medical devices?
(c) What firmware, version, operating system, software, or applications are utilized in manufacturing medical devices?
(d) What kind of vulnerabilities are potentially presented in specific IoMT devices?
(e) Are any of the medical devices vulnerable in a remote patient monitoring setting?
(f) Which type of data is more sensitive to vulnerabilities/attacks?
(g) Which population is more affected? i.e., patients, doctors, administration, etc.
(h) What are the potential consequences and adverse side effects of identified vulnerabilities/attacks?
(i) What countermeasures should be implemented for specific vulnerabilities?
(j) *Ontology exclusion:* Which medical devices or vulnerabilities are not addressed in this ontology?

#### 4.1.3. Answers to the Competency Questions

The IoMT environment integrates a range of devices, including actuators and medical sensors, facilitating remote patient monitoring. In our analysis, we identify vendors supplying medical devices crucial for remote monitoring, along with their specific configuration details. Notably, we exclude devices primarily utilized in clinical or hospital settings, such as surgical instruments. Utilizing ontology, we conduct a thorough examination to assess the vulnerability status of these medical devices while also evaluating potential consequences. Consequently, we propose tailored countermeasures to address identified vulnerabilities accordingly.

### 4.2. Reusing Existing Ontologies

Reusing existing ontologies can significantly reduce the time and effort required for development and ensure alignment with industry standards. The choice of the most suitable ontology development approach depends on defining the starting point: whether to build the ontology by integrating existing ones or to create it from scratch [32]. Several existing ontologies in the domain of healthcare, medical devices, and cybersecurity were examined that could potentially be reused or serve as a foundation for developing a new ontology.

Our literature review indicates that existing security ontologies are typically either exclusively focused on vulnerabilities or cyberthreat aspects (IDS [28], IoTSec [30], UCO [40]) and rarely, if at all, integrate cybersecurity-health interactions. Given the complexity and importance of these interactions in impact propagation studies, their formalization cannot be limited to merely combining the two separate domains. Moreover, the healthcare ontologies SNOMED CT [11], SafeCareOnto [48], and Medical Device Ontology (MDO) [40], among others, generally emphasize medical process terminologies or hospital settings and frequently neglect security considerations for remote patient monitoring environments.

### 4.3. The Term Enumeration

In the context of ontology engineering, the term ‘enumeration’ typically refers to a concept in which a specific property or attribute can take on one of a limited, defined set of values. These values are explicitly listed, and no other values are permitted for that property. Enumeration is a powerful tool in ontology engineering as it provides clarity and structure to the data model, ensuring that the ontology accurately represents the real-world domain it is intended to describe [37].

In the context of IoMT and cybersecurity, a few examples of terms used for MIoT ontology are: class (MedicalDevice, Vendor, Product, Person, Vulnerability, Exploit), subclasses (Actuators, MedicalSensor, Patient, Doctor, HealthcareProvider), Object properties (hasProduct, hasVenor, hasVulnerability, hasExploit, moniterdBy, effects), and data properties (hasName, hasSeverity, hasPublished). The next steps are developing a class hierarchy and defining the properties of these classes (concepts), called conceptualization.

### 4.4. Conceptualization

The conceptualization phase is very important in any software engineering development process. It defines the principles and design criteria to develop the domain ontology. Conceptualization involves organizing domain knowledge into a conceptual model that describes the problem and its solution using the vocabulary of the specific domain. By structuring information in this way, it becomes easier to understand and communicate complex concepts within the domain.

There are various approaches to developing a class hierarchy. Selecting the most appropriate approach for ontology development depends on determining the starting point, which involves deciding whether to build the ontology by integrating existing ontologies, reusing those ontologies, or creating it from scratch. As discussed in Section 4.2, the existing ontologies are not within our domain of scope, so we have adopted the scratch method to align with our project specifications. The ‘from scratch’ method [49,50] includes top-down, bottom-up, and middle-out approaches. Top-down approaches entail identifying a core of abstract, generic concepts and expanding it through specialization into more domain-specific concepts. In contrast, bottom-up approaches involve capturing task specifications, gathering terms, and defining new concepts at a low level. These low-level concepts can later be generalized to a higher level. Middle-out approaches establish core concepts before deriving new ones through specialization and generalization based on expert data.

The top-down approach is used for ontology development in our work. The concepts, relations, and axioms are the primary elements. Various types of links connect concepts at higher levels, including equivalence links (synonyms), hierarchical links (inheritance), and associative links (associations/relationships). Each concept denotes a collection of distinct individuals, also referred to as instances. Axioms facilitate the definition of the semantics of concepts and relations, expressing certain constraints on their values or cardinalities. The incorporation of axioms allows for the representation of specific capabilities or features of a concept while preventing the introduction of new concepts that might not be reused [51]. This way, the information about IoMT, RPM, and vulnerabilities can be queried or updated by the corresponding people or applications. The conceptualization phase consists of a set of activities [43], such as:

(a) Identifying concepts and their instances, attributes, and corresponding values within a *Data Dictionary (DD)*.
(b) Organizing various concepts into hierarchical structures in *concept classification trees*.
(c) Detailing properties such as object properties and data properties of classes and instances in respective *Table of object properties* and *Table of data properties*.
(d) Recording specific instances in a *Table of Instances*.

#### 4.4.1. Data Dictionary

The creation of a Data Dictionary (DD) marks the initial step in capturing domain knowledge. This essential document identifies and gathers relevant domain concepts, including their meanings, properties, instances, and other relevant details [52]. Each concept in the domain may be documented with fields such as concept name, synonyms and acronyms, concept description, instances of the concept, class attributes, and instance attributes. The resulting Data Dictionary is a comprehensive reference for understanding the domain. A few high-level concepts from MIoT ontology for DD are provided in Table 4, with some of their instances, class attributes, and instance attributes.

#### 4.4.2. Concepts Classification Tree

**Figure 1:**
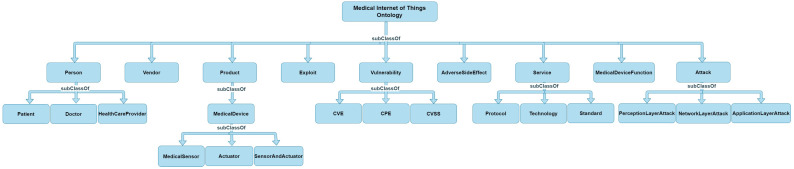
Class diagram for the concept classification trees.

After nearly completing the DD, the next step is to develop concept classification trees (CCTs). These trees are used in ontology building to organize domain concepts into taxonomies, forming a class/subclass hierarchy. These trees are not just for understanding relationships among concepts but also for dividing domain knowledge into independent, modular ontologies. The concepts described in DD must be shown in the CCTs and vice versa. Figure 1 shows the concept classification trees for MIoT ontology.

#### 4.4.3. Table of Object Properties and Table of Data Properties

Furthermore, object and data properties are two fundamental types of properties used to describe the relationships and attributes of individuals (instances) in a domain. These properties are commonly used in knowledge representation and Semantic Web technologies. Object properties express relationships between concepts or instances in the ontology, while data properties are used to assign values to individuals, representing attributes or characteristics of instances using literal values (strings, integers, etc.).

(a) Examples of Object Properties

:MedicalDevice rdf:type owl:Class.

:Vulnerability rdf:type owl:Class.

:hasVulnerability rdf:type owl:ObjectProperty.

In this turtle example, MedicalDevice and Vulnerability are concepts and hasVulnerability is an object property.

:InsulinPump rdf:type:MedicalDevice.

:Heartbleed a:Vulnerability.

:insulinPump:hasVulnerability:Heartbleed.

In this turtle example, InsulinPump and Heartbleed are instances of ontology and hasVulnerability is an object property. The full list of object properties with their domain and range is given in Table 5.

(b) Examples of Data Properties

:hasBloodPressure rdf:type owl:DatatypeProperty.

:BloodPressure rdf:type:MedicalDeviceFunction.

:BloodPressure:hasBloodPressure “120”^^xsd:int.

In this turtle example, BloodPressure is an individual and hasBloodPressure is a datatype property, and BloodPressure has a blood pressure value of at least 120.

:hasSeverity rdf:type owl:DatatypeProperty.

:RemoteCodeExecution rdf:type:Vulnerability.

:RemoteCodeExecution:hasSeverity “High”^^xsd:string.

In this turtle example, RemoteCodeExecution is an individual and hasSeverity is a datatype property, and RemoteCodeExecution has a high severity.

In these examples, RDF syntax is used to illustrate how ontological concepts can be represented. The terms like rdf:type, owl:Class, owl:ObjectProperty, and owl:DatatypeProperty come from OWL, which is commonly used for creating ontologies. The list of a few data properties with their domain and range is shown in Table 6. These data properties have been selected based on their relevance to the competency questions and their widespread usage in the fields of IoMT and cybersecurity.

#### 4.4.4. Table of Instances

Based on the scope and domain of the ontology, and after defining the concepts and relationships, gathering instances that represent these concepts in the real world is important. These instances should be diverse yet typical, covering various aspects of the domain. Additionally, ensuring the relevance of selected instances is essential; they should accurately illustrate the characteristics and behaviors of the concepts they represent. Considering the potential use case or application of the MIoT ontology, such as for vulnerability detection in remote patient monitoring, the instances are defined to demonstrate the functionality and utility of the ontology in practical scenarios.

After finalizing all instances (individuals) listed in the ‘Instance’ field of the Data Dictionary (DD) within the domain, the subsequent step involves creating a table of instances for each instance identified in the DD. An example of such a table is illustrated in Table 7. This process involves the instance name, description, and related concepts.

### 4.5. Formal Grounding with Description Logics

Formal grounding is a subsequent process that involves explicitly and logically defining concepts (classes), roles (relationships), and individuals (instances). In the realm of knowledge representation, formal grounding ensures the consistency, logic, and utility of the ontology for inference purposes. This approach facilitates sophisticated reasoning and querying capabilities within ontologies [34,53,54]. The hierarchical arrangement within the knowledge domain of the ontology, shown in Figure 1, has been formally established using a description logic formalism with an expressivity of *ALO^(D)^*. Some examples of concepts, roles, and individuals within the ontology comprise the following three headings:

#### 4.5.1. Concepts Hierarchy in Description Logic

One fundamental aspect of formal grounding is the development of a concept hierarchy using description logic (DL) axioms. This hierarchy organizes concepts in a structured set of concepts (classes) and their relationships in a formal, logical manner. In the context of ontologies, a concept hierarchy is a way of organizing knowledge where more general concepts are at higher levels and more specific concepts are at lower levels. In DL, this is primarily achieved using subclass relationships. Key elements in concept hierarchy with DL axioms are given here:

- Concepts (Classes): In DL, concepts are the basic units and represent sets of individuals (or objects). They are denoted by capital letters and singular. For example, Vulnerability, Person, and Actuator are shown as an example in Figure 1.
- Subclass relation: The subclass relation ⊑ is used to define a hierarchy between concepts. If A ⊑ B, it means every instance of concept A is also an instance of concept B. For instance, MedicalSensor ⊑ MedicalDevice indicates that medical sensors are medical devices.
- Top and bottom concepts: Top (⊤) or universal concepts and bottom (⊥) concepts are used to denote the broadest and the most restricted scopes, respectively. For example, ⊤ could be used to denote a concept that is too general or unspecific, and it includes all individuals. Meanwhile, ⊥ can be used to represent an impossible or contradictory concept and includes no individuals.

Concept hierarchy is started by defining basic concepts such as:

MedicalDevice, Vulnerability, Exploit, Target, Person etc.

Specific concepts are then created and defined in their relationship to the general ones by defining subclass relationships such as:

Patient ⊑ Person, MedicalDevice ⊑ Product, MedicalSensor ⊑ MedicalDevice, Technology ⊑ Service etc.

Furthermore, define concepts with restrictions, like property restrictions or cardinality constraints, for instance.

MedicalDevice ⊑∃ usingConnectivity. Technology

This axiom shows that ‘Every instance of MedicalDevice is associated with at least one instance of Technology through the usingConnectivity property.’, where

‘MedicalDevice’ refers to the class of Product.

‘⊑’ denotes subclass or subclass relationship.

‘∃’ denotes existential quantification, meaning ‘there exists’.

‘usingConnectivity’ is a property that relates a medical device to a technology.

‘Technology’ refers to the class of service.

#### 4.5.2. Role Definition in Description Logic

In DL, these properties are called roles, i.e., object role and data role. An object role is typically defined as a binary relation. For example, if R is an object role and a and b are individuals, then R(a, b) means that there is a relationship R between a and b. In MIoT ontology, e.g., hasVulnerability (bloodPressureMonitor, heartBleed), hasVulnerability is an object, i.e., R, and bloodPressureMonitor and heartBleed are individuals. It shows that bloodPressureMonitor has a vulnerability named heart bleed. Meanwhile, a data role links an individual to a data value. For example, if D is a data role, a is an individual, and v is a data value, then D(a, v) indicates that the individual a has a data property D with value v. For instance, monitor (Pacemaker, HealthCondition) would mean that Pacemaker is used to monitor health condition. Roles can be restricted using existential quantification (∃R.C, meaning there exists a relationship R to an individual of class C) or universal quantification (∀R.C, meaning all relationships R lead to individuals of class C). For example, existential quantification ∃ in formal logic, shown as

MedicalDevice ⊑∃ hasVulnerability. Vulnerability.

It shows that every instance of MedicalDevice is associated with at least one instance of Vulnerability through the role hasVulnerability.

In turtle format:

:MedicalDevice rdf:type owl:Class.

:Vulnerability rdf:type owl:Class.

:hasVulnerability rdf:type owl:ObjectProperty.

:MedicalDevice rdf:subClassOf [

rdf:type owl:Restriction;

owl:onProperty:hasVulnerability;

owl:someValuesFrom:Vulnerability].

Similarly, universal quantification ∀ in formal logic is shown as:

MedicalDevice ⊑∀ hasExploit. Exploit.

It shows every instance of MedicalDevice is associated with only instances of Exploit through the role hasExploit.

In turtle format:

:MedicalDevice rdf:type owl:Class.

:Exploit rdf:type owl:Class.

:hasExploit rdf:type owl:ObjectProperty.

:MedicalDevice rdf:subClassOf [

rdf:type owl:Restriction;

owl:onProperty:hasExploit;

owl:allValuesFrom:Exploit].

#### 4.5.3. Individual Declarations

In DL, the formal grounding of individuals provides a foundation for accurately and consistently representing specific entities and their relationships within an ontology. In the context of formal grounding, individual-related assertions following the syntax and semantics of description logic, such as:

MedicalSensor(pacemaker) states that pacemaker is an individual of the class MedicalSensor.
In another axiom, monitor(pacemaker, healthCondition) could represent that pacemaker monitor health condition.Assertions can also be more complex, involving roles and role restrictions. For example,
∃monitor.MedicalSensor(pacemaker) indicates that the pacemaker monitors at least one object of the class MedicalSensor.

Based on the defined individuals and their relationships, the reasoning engine infers new knowledge, checks for inconsistencies, and answers complex queries.

## 5. Developing the Conceptual Model for MIoT Ontology

The conceptual model for MIoT ontology, named the MIoT model, provides a centralized repository and utilities that all participants in this system can share and communicate effectively. This will speed up the development process, foster interoperability, and improve the correlation of test results. The model is based on the proposed methodology (KEM), where concepts, their properties, and relationships are defined.

### 5.1. Detailed Description of MIoT Model

**Figure 2:**
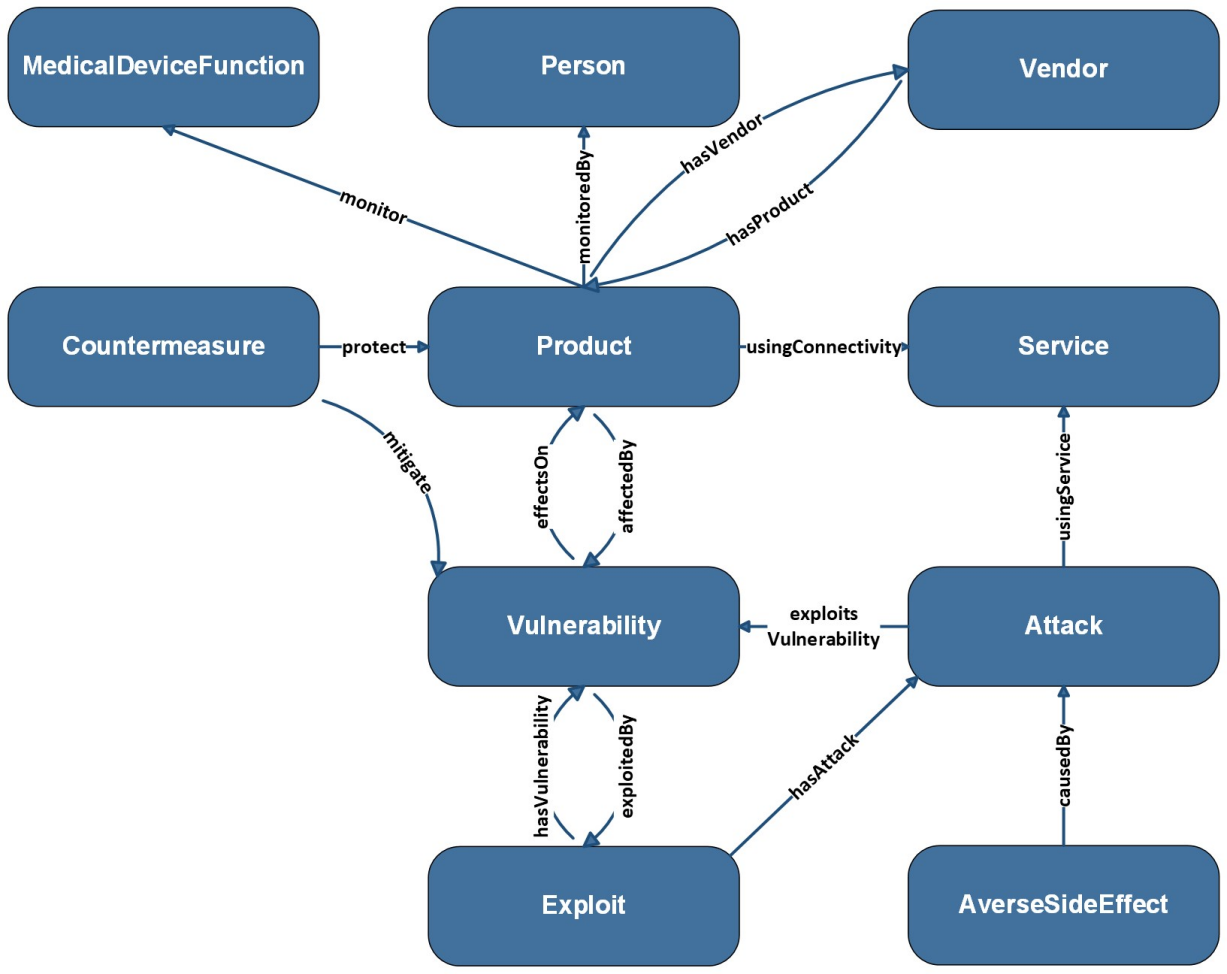
The Conceptual model of MIoT ontology.

The main ideas in MIoT (Medical Internet of Things) ontology are interconnected (concepts) through various properties (relationships), serving different purposes within the scope of the Internet of Medical Things (IoMT) and addressing various scenarios and use cases. For example, a medical device (such as an insulin pump) monitors a device function (such as glucose level), manufactured by a vendor (such as XYZ) and monitored by a person (such as the patient). A vulnerability that exists in the product (such as the Heartbleed Vulnerability), can be exploited by an attack (such as a DDoS attack) due to using a service (such as Wi-Fi), leading to adverse effects. Countermeasures can then be applied to protect the product by mitigating the vulnerability. This is a simple use case addressed by our model. Researchers can perform rule-based reasoning on the proposed model to address more comprehensive use cases. Figure 2 below illustrates the MIoT model where each high-level concept and their related properties are described.

**Figure 3:**
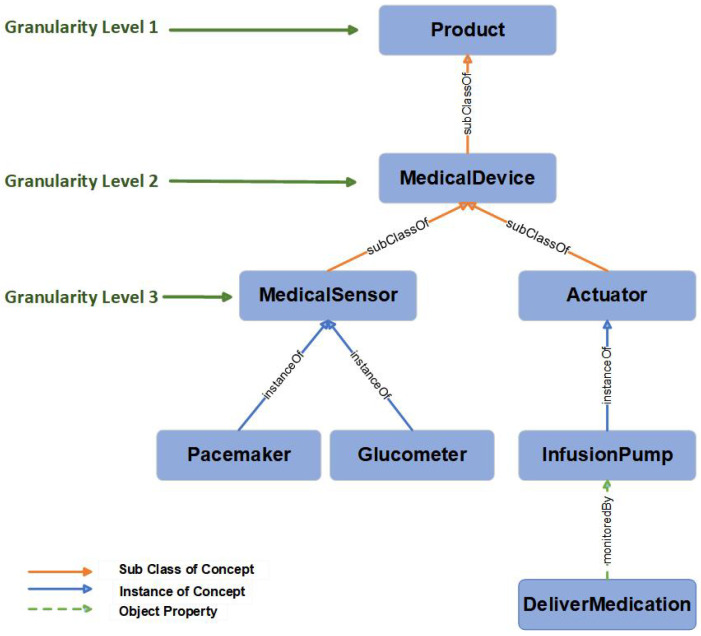
The granularity of MIoT ontology described for the concept *Product* with subclasses, instances, and relationship.

The MIoT ontology is designed to articulate concepts not only at a high level but also to reach deeper level, spanning across three distinct levels of granularity. At Level 1, foundational concepts such as Product, Vulnerability, Exploit, Person, and Service are established. Level 2 extends this framework to include domain-specific entities, listed as terms such as Medical Device, Patient, Healthcare Provider, and Technology. Finally, Level 3 refines the ontology with specialized subclasses such as Medical Sensor and Actuator. A detailed illustration is shown in Figure 3 describing the hierarchical design of the concept product with its subclasses across these three levels. It is worth noting that the lower-level occurrences are facilitated through rule-based reasoning, which are security elements that fall beyond the scope of this current paper. Fundamental expressions such as ‘product’, ‘medical device’, ‘patient’, and ‘healthcare provider’ describe the IoMT entities, while terms such as ‘vulnerability’, ‘exploit’, ‘attack’, and ‘countermeasure’ define the crucial roles that characterize the various security vulnerabilities. Details about descriptions, properties, and instances are discussed in the Data Dictionary table.

### 5.2. Case Study

To define the problem domain clearly and discuss the concepts, relationships, and constraints involved in the MIoT ontology and evaluate the performance, a hypothetical scenario is presented here, which is adopted from the Digital Living Lab [55].

Suppose we have scenario *X*; John, a patient managing hypertension. He relies on a comprehensive remote patient monitoring system, which includes a blood pressure monitor and a diabetes management tool. However, upon noticing alarming blood pressure readings, he alerts his doctor, leading to the discovery of a network vulnerability. An attacker exploited this vulnerability, compromising the accuracy of John’s readings and causing serious risks to his health. The doctor immediately takes steps to address the issue and ensure that John’s monitoring system is secure. They also advise John to continue monitoring his blood pressure manually until the issue is resolved.

Despite these efforts, further complications arise when John’s infusion pump used for medication administration is also compromised weeks later. An attacker gains access to the pump’s network connection, altering medication dosages and resulting in severe adverse effects for John, requiring hospitalization. This incident underscores the critical need for secure remote patient monitoring systems, emphasizing the imperative of protecting medical devices from vulnerabilities and ensuring patient safety. There is a need to automate the system that takes security measures and alarms the patient and healthcare providers about the vulnerabilities in the system. It must be implemented to mitigate risks associated with vulnerabilities exploited by attacks, safeguarding the accuracy and integrity of medical device data transmission through the network.

In terms of vulnerability detection, this work develops assumptions based on scenario x. The generated assumptions from the provided hypothetical scenario would focus on vulnerability identification and its consequences on patients, medical devices, and data and propose countermeasures to mitigate attacks that exploit vulnerabilities.

#### Assumptions

1. Medical devices for remote patient monitoring systems from a Vendor utilize network connectivity to transmit data to healthcare providers.
2. Due to the attack, data are modified and transmitted between the medical device and the healthcare provider through the network layer, compromising the confidentiality and integrity of the patient’s information.
3. Vulnerabilities in the network layer of remote patient monitoring systems may lead to inaccurate readings or manipulation of medical data.
4. The compromise of medical devices, such as infusion pumps, due to vulnerabilities in their network layer, can have severe consequences, including adverse side effects for patients and the need for hospitalization to address medication-related issues.
5. The incident with John’s infusion pump underlines the importance of implementing robust security measures in remote patient monitoring systems to safeguard against potential attacks and vulnerabilities that could risk patient safety and compromise the accuracy of medical data.

### 5.3. Evaluation

Identifying potential weaknesses and vulnerabilities during the design phase is crucial for establishing robust security measures from the beginning. Formal methods offer a promising way to determine such flaws at an early stage. Through formal verification and validation techniques, reliability and security can be assured, assessing the accuracy of designs using diverse mathematical and logical methods [56,57]. Furthermore, formal methods identify design errors early in the development process and ensure that the system aligns with specifications, requirements, and standards [58]. Formal methods, such as mathematical and logical notations, employ automated tools to analyze the behavior and properties of the system, including tasks such as checking for consistency, completeness, and correctness [59]. In our research, we use logical methods, more specifically, symbolic logic (i.e., propositional logic). In propositional logic, axioms are statements that define relationships or properties within an ontology to satisfy the given assumptions. We also define the notations that represent the concepts in MIoT ontology, as shown in Table 8.

For the given assumptions, we write the axioms in the form of propositional logic. Additionally, notations are defined to represent the concepts of MIoT ontology in Table 8.

**Assumption** **1.** *These axioms define the relationships between the medical devices, vendors, network connectivity through the network layer, and healthcare provider in the context of the remote patient monitoring system.*

*manufacturedBy(Med1, V) ∧ manufacturedBy(Med2, V)*

*usingConnectivity(Med1, NL) ∧ usingConnectivity(Med2, NL)*

*receiveData(HP, NL)*

**Assumption** **2.** *These axioms define the relationships between attack, medical device, network connectivity, healthcare provider, and patients’ information, specifying the actions that attackers may take and their potential consequences in compromising the integrity and confidentiality of patients’ information.*

*Attack(A) ∧ interceptsData(A,MD,HP) ∧ hasModifiedData(NL,true)*

*→ compromisesIntegrity(P) ∧ compromisesConfidentiality(P)*

**Assumption** **3.** *These axioms define the relationships between vulnerabilities in the network layer, inaccurate readings, or manipulation of medical data, specifying the potential consequences of network vulnerabilities in the remote patient monitoring system.*

*Vulnerability(Vul) ∧ inNetworkLayer(V,NL)*

*→ (inaccurateReadings(NL) ∨ manipulationOfMedicalData(NL))*

**Assumption** **4.** *This axiom states that if a medical device has a vulnerability and is connected to a network layer, then this vulnerability can lead to adverse side effects for patients and requires hospitalization to address medication-related issues.*

*MedicalDevice (MD) ∧ hasVulnerability(MD,Vul) ∧ connectedTo(MD,NL)*

*→ causedAdverseSideEffect (A,ASE) ∧ requiresHospitalization(ASE,H)*

**Assumption** **5.** *If there is an incident with John’s infusion pump, this axiom emphasizes the importance of taking countermeasures to implement robust security measures, which in turn helps prevent potential attacks and ensures the accuracy and integrity of medical data in remote patient monitoring systems.*

*takeCountermeasure(C) → implementRobustSecurityMeasures(C) ∧*

*preventAttack(MD2,C) ∧ preventInaccurateReadings(NL,C)∧*

*preventManipulationOfMedicalData(NL,C)*

In summary, the evaluation of this case study demonstrates that the proposed model strongly aligns with a system that addresses the key concerns relevant to the areas of interest in this solution. The case study illustrates the model’s effectiveness in addressing critical issues such as confidentiality and integrity. It indicates that assumptions based on a logic-based approach are effective in detecting and responding to key vulnerabilities, especially those occurring at the network layer, as evidenced by the majority of attacks in the presented case study.

## 6. Limitations and Future Work

This MIoT model aims to identify security issues, such as vulnerabilities, in the IoMT scenario from the perspectives of patients, doctors, and healthcare providers. While it is acknowledged in different literature [60,61,62] that caregivers are also part of IoMT, this aspect is not addressed in this work. Our focus is on those who directly interact with IoMT devices. To address this limitation, future work will include caregivers or other related stakeholders, such as pharmacists and accountants in the IoMT domain. Another challenge faced by researchers is the lack of publicly available datasets or real-time sensor data that address security issues such as vulnerabilities or cyberattacks in IoMT. To cope with this challenge, this work adopted a case study approach to validate our work.

The MIoT ontology has significant potential to revolutionize security automation within healthcare applications by providing a structured framework for representing and understanding the relationships among medical devices, vulnerabilities, and network infrastructure. Building upon the predefined concepts and relationships within the MIoT ontology, we aim to introduce automation capabilities within MIoT ontology through case studies for vulnerability analysis and reasoning. For this purpose, we will establish rules to enable automated reasoning to make more effective inferences and high-level decisions. To evaluate our results, the standardized vulnerability information provided by NVD in terms of CVE and CVSS (Common Vulnerability Scoring System) will be integrated with the MIoT ontology for the assessment and management of security vulnerabilities across various medical devices. This conceptual model will be implemented, visualized, and queried in a knowledge graph to store CTI (cyberthreat intelligence) for semantic representation and data integration.

## 7. Conclusions

An ontology serves as a structured and formal representation of knowledge, providing enhanced tools for communication, reusability, and knowledge organization. Semantic technologies not only facilitate communication and representation but also lay the groundwork for reasoning and decision making by enabling computers to understand the meaning (semantics) behind data. The focus of this research paper is to develop the MIoT ontology, which captures knowledge from two cross domains (i.e., IoMT and Cybersecurity) and facilitates the interconnections between remote patient monitoring and security infrastructure, where vulnerability detection takes place. While numerous publications explore into the application of semantic technologies in cybersecurity and healthcare, to the best of our knowledge, the MIoT ontology is a novel ontology expressly crafted for vulnerability detection in the remote patient monitoring setting. This ontology outlines fundamental concepts for vulnerability detection in remote medical devices, explaining their properties and defining their relationships through the utilization of knowledge engineering methodology. To add weight to our overall conclusion regarding the effectiveness of this proposed solution, the MIoT model underwent validation against a representative case study, demonstrating strong alignment with the model’s vulnerability detection through the formal logic of an axiom-based approach.

## Figures and Tables

**Table 1:** FOL, DL, and OWL Terminologies.

FOL	DL	OWL
unary predicate	concept	class
binary predicate	role	property
constant	individual	individual

**Table 2:** Definition of terms used in description logic (DL).

Abbreviations	Stands for	Description
ABox	Assertional Box	A component that holds statements about individuals, such as OWL facts including type declarations, property-value relationships, as well as assertions of equality or inequality.
TBox	Terminological Box	A component that stores statements about classes, including OWL axioms like subclass relationships, equivalent classes, or disjointedness declarations.
KB	Knowledge Base	A combination of an ABox and a TBox represents an ontology.

**Table 3:** Ontology development.

Stages for Ontology Development
Specification and Scope
Reuse Existing Ontologies
Term Enumeration
Conceptualization
Formal Grounding with Description Logics

**Table 4:** Data Dictionary for the concepts of *Product, Vendor, Service, and Vulnerability*.

Concept Name	Description	Instances	Class Attributes	Instance Attributes
Product	Refers to medical devices or other healthcare products	Medfusion4000Pump, CRH ^1^, GMS ^2^	affectedBy, hasVendor, monitor, attackedBy, monitoredBy, purchasedBy, usingCoonnectivity	hasVersion, hasName, hasSensorType, productID
Vendor	An entity that sells or supplies medical devices to healthcare organizations, hospitals, clinics, or patients	Abbott, SmithMedical, IHealth, MySignals, Foracare, BostonScientific, AliveTech	hasProduct, hasVendor	hasName, manufacturedID
Service	It is a communication pathway to transfer data between two devices	Bluetooth, CloudBased, Wi-Fi, MQTT, BLE	usingConnectivity, disruptsService targetService byUsingService	hasBluetooth, hasName
Vulnerability	A vulnerability refers to a weakness or flaw in a system’s design, implementation, or configuration that could be exploited by attackers to compromise the security of the system, its data, or its users	RemoteCodeExecution, BufferOverflowVulnerability	hasVulnerability, exploitedBy, effectsOn, mitigate	vulnerabilityID, vulnerabilitySeverity, VulnerabilityVersion, hasImpactScore, hasExploitabilityScore, hasAvailabilityImpact, hasConfidentialityImpact, hasIntegrityImpact, hasSeverity

^1^ CardiacRhythmManagement. ^2^ GlucoseMonitoringSystem.

**Table 5:** Table of object properties with their domain and range.

Object Properties	Domain	Range
affectedBy	Product	Vulnerability
attackedBy	Product	Attack
byUsingService	Service	Person
causedBy	AdverseSideEffect	Attack
disruptsService	Attack	Service
effectsOn	Vulnerability	Product
exploitedBy	Vulnerability	Exploit
exploitsVulnerability	Attack	Vulnerability
hasAttack	Exploit	Attack
hasCredential	Patient	Product
hasProduct	Vendor	Product
hasVendor	Product	Person
hasVulnerabilty	Exploit	Vulnerability
Mitigate	Countermeasure	Vulnerability
Monitor	MedicalDevice	MedicalDeviceFunction
monitoredBy	Product	Vendor
Protect	Countermeasure	Product
purchasedBy	Product	Patient
targetService	Attack	Service
usingConnectivity	Product	Service
usingDevice	Patient	Product
usingService	Patient	Service

**Table 6:** Table of data properties with their domain and range.

Data Properties	Domain	Range
attackType	Attack	String
hasAccessComplexity	Vulnerability	String
hasAccessVector	Vulnerability	String
hasAttack	Attack	String
hasAuthentication	Vulnerability	String
hasAvailabilityImpact	Vulnerability	String
hasBaseScore	Vulnerability	String
hasBloodPressure	MedicalDevice	Integer
hasBluetooth	Technology	String
hasDosageValue	MedicationDosage	Decimal
hasModifiedData	NetworkLayer	Boolean

**Table 7:** Table of instances and their description.

Instance Name	Instance of	Description
Medtronic ^1^	Vendor	is a vendor of medical devices
BloodPressureMonitor	SensorAndActuator	is a medical device used to measure and monitor a person’s blood pressure
BufferOverflowVulnerability	Vulnerability	is a type of security flaw or weakness in a computer program
CardiacRhythmManagement	Product	are designed to diagnose, manage, and treat disorders related to heart
HealthCondition	MedicalDeviceFunction	refer to a condition related to health
InfusionPump	Actuator	utilized to introduce fluids, medications, or nutrients into the circulatory system of a patient.
LifeThreatening	AdverseSideEffect	a situation where a danger occurs to someone’s life.
MQTT	Protocol	is a messaging protocol and well-suited for communication between devices with limited resources, such as sensors and actuators.
Wi-Fi	Technology	is a set of wireless communication standards used for transmitting data between devices over short distances such as medical devices.

^1^ https://www.medtronic-diabetes.com.au/ [Accessed on 10 March 2022].

**Table 8:** Notations for concepts used in scenario *X*.

Notations for Concepts	Description
A	Attack
ASE	Adverse side effect
C	Countermeasure
H	Hospitalization
HP	Healthcare provider
MD	Medical device
MD1	Type of medical device (i.e., BP monitor)
MD2	Type of medical device (i.e., Infusion pump)
NL	Network layer
P	Patient
V	Vendor
Vul	Vulnerability

## Data Availability

Data are contained within the article.
